# Polyphenols and Oxidative Stress in Atherosclerosis-Related Ischemic Heart Disease and Stroke

**DOI:** 10.1155/2017/8526438

**Published:** 2017-11-26

**Authors:** Yu-Chen Cheng, Jer-Ming Sheen, Wen Long Hu, Yu-Chiang Hung

**Affiliations:** ^1^Department of Chinese Medicine, College of Medicine, Kaohsiung Chang Gung Memorial Hospital and Chang Gung University, Kaohsiung, Taiwan; ^2^Kaohsiung Medical University College of Medicine, Kaohsiung, Taiwan; ^3^Fooyin University College of Nursing, Kaohsiung, Taiwan; ^4^School of Chinese Medicine for Post Baccalaureate, I-Shou University, Kaohsiung, Taiwan

## Abstract

Good nutrition could maintain health and life. Polyphenols are common nutrient mainly derived from fruits, vegetables, tea, coffee, cocoa, mushrooms, beverages, and traditional medicinal herbs. They are potential substances against oxidative-related diseases, for example, cardiovascular disease, specifically, atherosclerosis-related ischemic heart disease and stroke, which are health and economic problems recognized worldwide. In this study, we reviewed the risk factors for atherosclerosis, including hypertension, diabetes mellitus, hyperlipidemia, obesity, and cigarette smoking as well as the antioxidative activity of polyphenols, which could prevent the pathology of atherosclerosis, including endothelial dysfunction, low-density lipoprotein oxidation, vascular smooth muscle cell proliferation, inflammatory process by monocytes, macrophages or T lymphocytes, and platelet aggregation. The strong radical-scavenging properties of polyphenols would exhibit antioxidative and anti-inflammation effects. Polyphenols reduce ROS production by inhibiting oxidases, reducing the production of superoxide, inhibiting OxLDL formation, suppressing VSMC proliferation and migration, reducing platelet aggregation, and improving mitochondrial oxidative stress. Polyphenol consumption also inhibits the development of hypertension, diabetes mellitus, hyperlipidemia, and obesity. Despite the numerous *in vivo* and *in vitro* studies, more advanced clinical trials are necessary to confirm the efficacy of polyphenols in the treatment of atherosclerosis-related vascular diseases.

## 1. Introduction

Atherosclerosis-related ischemic heart disease (IHD) and stroke are the leading cause of morbidity or mortality worldwide for decades [1, 2]. Oxidative stress [3–5] is found to be associated with some risk factors [6, 7] of atherosclerosis, such as hypertension, diabetes mellitus, hyperlipidemia, obesity, and cigarette smoking. Numerous relevant studies investigating disease ontology and seeking for effective diagnostic measures and therapies exist. Some researchers found that nutrient antioxidants could help inhibit atherosclerosis process [8–11]. Following, we make a brief introduction of oxidative stress and polyphenols.

### 1.1. Oxidative Stress

Reactive oxygen species (ROS) are generated as metabolic by-products by biological systems, including superoxide radicals (•O_2_^−^), hydrogen peroxide (H_2_O_2_), and hydroxyl radicals (•OH) [12]. Nitric oxide (NO) plays an important role in vessel dilatation and inflammation. Normally, NO is produced by endothelial nitric oxide synthase (eNOS) in the vessel endothelium. But in the inflammatory process, inducible nitric oxide synthase (iNOS) expresses in macrophages and smooth muscle cells and also produces NO. When •O_2_^−^ contacts to NO, they rapidly react to form the highly reactive molecule peroxynitrite (ONOO^−^). •O_2_^−^ is rapidly dismutated to the more stable ROS, H_2_O_2_, by superoxide dismutase (SOD), which is then converted to H_2_O and O_2_ by either catalase or glutathione peroxidase [13].

ROS [14] may also play a vital role in the progressive pathology of atherosclerosis, which involves endothelial dysfunction, oxidized low-density lipoprotein (OxLDL) [15], vascular smooth muscle cell (VSMC) proliferation, inflammatory process by monocytes, macrophages, or T lymphocytes, and platelet aggregation. ROS origin from a variety of sources such as NO synthase (NOS), xanthine oxidases, the cyclooxygenases, nicotinamide adenine dinucleotide phosphate (NADPH) oxidase isoforms, and metal-catalyzed reactions [16]. Low-density lipoprotein (LDL) activates endothelial NADPH oxidase, predominantly through a signaling pathway that leads to cytosolic phospholipase A2 (PLA2) activation [17] and promoting ROS formation. Once ROS-induced OxLDL crossed the damaged endothelium into the intima, monocytes differentiate into macrophages, which would in turn take up OxLDL and subsequently become foam cells. These lipid-containing foam cells in the arterial wall can evolve into atherosclerotic plaques or atheromas. Ruptured plaques could result in IHD, stroke, and even death. Hence, reducing the mortality rate due to atherosclerosis is crucial.

### 1.2. Polyphenol

Polyphenols are common nutrient antioxidants, mainly derived from fruits, vegetables, tea, coffee, cocoa, mushrooms, beverages, and traditional medicinal herbs such as *Salvia miltiorrhiza* [18–20]. The classification of polyphenols mainly includes flavonoids (60%), phenolic acids (30%), and other polyphenols (including stilbenes and lignans) [21], attached at least one aromatic ring with one or more hydroxyl functional groups [22]. Flavonoids, the most studied group of polyphenol, are divided into six subclasses: flavonols, flavones, flavanones, flavanols, anthocyanins, and isoflavones. Phenolic acids are divided into two subclasses, benzoic acid and cinnamic acid. Stilbenes in plants act as antifungal phytoalexins and are rare in human diet. Resveratrol, found in grapes and red wine, is the well-known polyphenol in stilbene group. Lignans are rich in flax, sesame, and many grains [23, 24].

The bioavailability of polyphenols predominantly depends on gut microflora activity [25]. After intake, polyphenols are subjected to three main types of conjugation: methylation, sulfation, and glucuronidation. These metabolic reactions contribute to polyphenols' chemopreventive activities [22]. Polyphenols clearly improve the status of different oxidative stress biomarkers [25]. Previous studies had noted that flavonoids could scavenge superoxide anion and peroxynitrite. They also would exert some antioxidative activity by effectively regulating oxidative stress-mediated enzyme activity [26] and by chelation of the transition metals involved in radical-forming processes [27]. Through direct interactions with receptors or enzymes involved, cells respond to polyphenols which may trigger a series of redox-dependent reactions and result in modification of the redox status of the cells [28–30].

Polyphenols are potential substances against cancers and cardiovascular, metabolic [31], and neurodegenerative diseases [32] through their abilities of antioxidation and antimutation. The metabolism of polyphenols can neutralize free radicals by donating an electron or hydrogen atom to suppress the generation of free radicals or deactivate the active species and precursors of free radicals. Polyphenols, as metal chelators, chelate metal transition such as Fe^2+^ and directly reduce the rate of Fenton reaction, thus preventing oxidation caused by highly reactive hydroxyl radicals (•OH) [33, 34]. As the well antioxidative abilities of polyphenols, they may play important roles and interact with some cell receptor and intracellular signaling and/or gene expression regulation during atherosclerotic progressions. This review aims to present a novel focus on the role of antioxidative polyphenols and oxidative stress in atherosclerosis-related IHD and stroke.

## 2. Materials and Methods

The current review focuses on the role of polyphenols and oxidative stress in atherosclerosis-related IHD and stroke. The keywords were entered “polyphenol and oxidative stress and atherosclerosis, or polyphenol and oxidative stress and ischemic heart disease, or polyphenol and oxidative stress and stroke.” Literature searches were performed using the Medicine, PubMed, EMBASE, Cochrane library, CINAHL, and Scopus databases. We exclude papers from nonabove databases or non-English-writing articles.

## 3. Results and Discussion

### 3.1. Polyphenols and Oxidative Stress Associated with Risk Factors of Atherosclerosis

#### 3.1.1. Hypertension

Hypertension is closely associated with atherosclerosis, which is related to IHD and stroke. One of the underlying mechanisms for the enhanced atherogenesis in hypertensive patients is oxidative stress [5]. Polyphenols from red wine reduce blood pressure elevations by increasing nitric oxide synthase (NOS) activity; decreasing end-organ damage, for example, myocardial fibrosis and aortic thickening; and decreasing protein synthesis in the heart and aorta [35, 36].

Angiotensin II (AngII) is a significant factor in blood pressure regulation and is also involved in the process of atherosclerosis and in the remodeling through repairing processes of the myocardium following myocardial infarction [37]. AngII-induced hypertension is associated with blunted endothelium-dependent vasodilation. The increasing ROS formation in the arterial wall through nicotinamide adenine dinucleotide phosphate (NADPH) oxidase activation via type 1 AngII receptors leads to increased oxidative stress [38]. Moreover, AngII also induces the migration and proliferation of cultured VSMCs [39] and increases cytosolic Ca^2+^ levels, which was found to stimulate the DNA-binding activity of the transcription factor nuclear factor kappa B (NF-*κ*B) in cultured human neutrophils [40]. Furthermore, polyphenols can block AngII-stimulated upregulation of several NADPH oxidase (NOX) subunits, including NOX 1 and p22phox (an essential component of NOX), and the associated oxidative stress [38]. Based on these mechanisms, some researches revealed that systolic blood pressure in hypertensive patients is improved after ingesting polyphenol-rich foods [41, 42]. Combining dietary flavonoids and a pharmacological antihypertensive therapy based on telmisartan or captopril may improve blood pressure, lipid profile, obesity, and inflammation in young hypertensive patients of both sexes [43].

In a randomized, single-blinded, and controlled trial with a 4-year follow-up, consumption of extravirgin olive oil significantly decreased diastolic blood pressure; however, no differences in systolic blood pressure changes were observed [44]. A similar result was also reported in a randomized, controlled study of tea flavonoids [45].

The relationship between oxidative stress and hypertension is noteworthy. Some animal studies have found that high blood pressure would be associated with increased oxidative stress [46]. However, the effects of polyphenol on blood pressure were still inconsistent [42, 47, 48]. Further clinical studies on polyphenol in hypertension will be necessary.

#### 3.1.2. Diabetes Mellitus

Increasing ROS levels are an important trigger for insulin resistance [49, 50]. Hyperglycemia induces oxidative stress in patients with diabetes, and the overproduction of ROS contributes to the development of cardiovascular diseases (CVD) [51]. In the presence of hyperglycemia, vascular remodeling is augmented by uncoupled eNOS [52], endothelial superoxide levels that inhibit vascular smooth muscle and Na^+^-K^−^-ATPase activity increase [53], and transient receptor potential cation channel subfamily V member 4 that regulates vascular function is downregulated [54].

Gut microbiota lipopolysaccharide (LPS) may translocate into the bloodstream and subsequently contribute to adipose tissue inflammation and oxidative stress, which in turn leads to insulin resistance [55]. Polyphenols reduce LPS proinflammatory action by increasing the production of adiponectin and peroxisome proliferator-activated receptor gamma (PPAR*γ*), which is known as a key anti-inflammatory and insulin-sensitizing mediators [56]. Moreover, LPS increases intracellular ROS levels and the expression of genes encoding ROS-producing enzymes, including NOX2, NOX4, and iNOS. Polyphenols reverse these effects and upregulate manganese superoxide dismutase (MnSOD) and catalase antioxidant enzyme gene expression [56].

Diabetic vasculopathy is characterized by abnormal angiogenesis [57]. Excessive concentrations of vascular endothelial growth factor (VEGF) and its receptor expressions drive angiogenesis and cause complications, such as increased tumor growth and atherosclerotic plaques. Polyphenol inhibits angiogenesis by downregulating VEGF [56]. For example, curcumin could inhibit VEGF expression in streptozotocin-induced diabetic retina [58, 59] and chlorogenic acid could reduce retinal vascular hyperpermeability and leakage on diabetic retinopathy through decreasing VEGF levels [60] in a rat model.

Diabetes is a metabolic disease, and some comorbidities are related to IHD and stroke, such as hyperlipidemia, obesity, and hypertension. One randomized, placebo-controlled, double-blind study revealed that taking polyphenol-rich dark chocolate is effective in improving triglyceride levels in hypertensive patients with diabetes and in decreasing blood pressure and fasting blood sugar [42]. In another study, after a short-term polyphenol-rich dark chocolate administration, a significantly increasing insulin sensitivity and decreasing blood pressure in healthy subjects were noted [48].

#### 3.1.3. Hyperlipidemia

Hyperlipidemia is the most important risk factor for atherosclerosis [61, 62]. Increased transcytosis of lipoproteins is the initial event in atherogenesis. ROS generated by activated inflammatory cells and the production of oxidized lipoproteins are key points for atherosclerotic plaque erosion and rupture [63]. Theaflavins may compete with nicotinamide adenine dinucleotide phosphate (NADPH) which is a substrate of b-ketoacyl reductase of fatty acid synthase. They could significantly reduce EGF-induced biosynthesis of triglycerides, cholesterol, and fatty acids through downregulating the epidermal growth factor (EGF) receptor/phosphatidylinositol-3-kinase (PI3K)/protein kinase B(Akt)/Sp-1 signal transduction pathways [64].

AMP-activated protein kinase (AMPK) is an essential therapeutic target for obesity [65]. Theaflavins may modulate AMPK and ROS pathways to inhibit acetyl-CoA carboxylase activities [66]. They could improve the activation of forkhead box O3A (FoxO3A) which is a common target transcription factor for AMPK signaling. Another, theaflavins may upregulate MnSOD against oxidative stress to alleviate atherosclerosis and diabetic nephropathy [67].

Because there are more thearubigins and theaflavins in black tea than in green tea, black tea extract could be more able to inhibit the emulsion of lipid droplets and reduce the surface area to decrease fat digestion [68, 69]. In the processes of lipid metabolism, black tea polyphenol also could inhibit pancreatic lipase to reduce lipids hydrolyzed and lipid absorption [70, 71].

Additionally, data show a good lipid excretion ability after polyphenol consumption. In subjects who had high-lipid diet, intake of polyphenol-enriched oolong tea increased lipid excretion in the feces [72]. Hosoyamada and Yamada reported that a combination of fish oil and apple polyphenol in rats with a high-cholesterol diet showed decreasing serum and liver lipid concentrations and decreasing serum oxidative stress and promoted fecal bile acid excretion [73].

#### 3.1.4. Obesity

Obesity is one of the most common nutritional diseases worldwide. According to the World Health Organization (WHO), obesity is a body mass index ≥ 30. In 2014, >1.9 billion adults, 18 years and older, were overweight. Of these, >600 million were obese [74]. Moreover, obesity could also lead to serious diseases, such as CVD, type 2 diabetes, and cancer [75]. A 10 kg higher body weight is associated with a 3.0 mmHg increase in systolic and 2.3 mmHg increase in diastolic blood pressure. These increases translate into an approximately 12% increased risk for coronary heart disease and 24% increased risk for stroke [76, 77]. An epidemiological study revealed that obesity elevates systemic oxidative stress in humans [78].

Obesity results from a lipid metabolic imbalance and leads to fat accumulation in adipose tissues [79]. The adipose tissue is a significant source of TNF-𝛼, IL-6, resistin, leptin, angiotensinogen, and adiponectin [80]. In adipocytes, oxidative stress induces the production of the abovementioned proinflammatory adipokines as well as leptin and resistin, which play a role in maintaining insulin resistance [49, 81]. The relationship between obesity and insulin resistance has been recognized for decades [82]. One potential strategy to reduce inflammation and insulin resistance is consuming polyphenol-rich foods, such as grapes or their by-products, which have anti-inflammatory properties [31].

Green tea polyphenols may reduce leptin levels in the subcutaneous tissue of high-fat-diet-induced obese rats [43]. On the contrary, they could increase percentage of fat-free mass and glutathione peroxidase protein expression and decreased percentage of fat mass, serum insulin-like growth factor I, leptin, adiponectin, and proinflammatory cytokines in obese rats [83]. Nevertheless, black tea with polyphenols is more effective in reducing body weight. They may inhibit lipid and saccharide digestion and absorption and reduce calorie intake [71]. The other articles revealed that black tea with polyphenols could promote lipid metabolism by activating AMPK, attenuating lipogenesis and enhancing lipolysis. They would lower lipid accumulation by suppressing the differentiation and proliferation of preadipocytes and by reducing oxidative stress [79].

#### 3.1.5. Cigarette Smoking

Cigarette smoking is associated with vascular endothelial dysfunction [84], which is primarily related to the ROS in tobacco smoke (TS) [85, 86], nicotine, and inflammation. Smoking enhances oxidative stress not only through ROS production but also through weakening of the antioxidant defense systems [87–89]. TS contributes to a proatherosclerotic environment by triggering a complex proinflammatory response and mediates the recruitment of leukocytes through cytokine signaling [90]. Thus, smokers are 2–4 times more likely to suffer from coronary heart disease and stroke [91–93].

Smoking could induce the differentiation of monocytes into macrophages and a strong vascular proinflammatory response through upregulating endothelial proinflammatory genes, increasing the levels of proinflammatory cytokines, and activating matrix metalloproteinase. Being a strong vascular inflammatory primer, TS can accelerate the dysfunction of blood-brain barrier (BBB) and the loss of cerebral blood flow such as during ischemic stroke [85]. TS-induced toxicity at BBB endothelial cells is strongly correlated with the tar and NO levels in the cigarettes rather than the nicotine content [86].

Cigarette smoking promotes glucose intolerance, increases the risk of developing type 2 diabetes mellitus, and thus is a leading high risk of cerebrovascular and neurological disorders like stroke via ROS generation, inflammation, and BBB impairment [94]. One trial revealed that metformin (an antidiabetic drug) activates counteractive mechanisms primarily associated with the nuclear factor erythroid 2-related factor pathway, which drastically reduces cigarette smoking toxicity at the cerebrovascular level [95].

Cigarette smoking causes oxidative stress, hypertension, and endothelial dysfunction. Polyphenol-rich foods, which are good antioxidants, could prevent these conditions. Antioxidant supplementation reduced the oxidation and inflammation induced by TS in animals and cells [94, 96]. One randomized controlled trial involving young volunteers demonstrated that blueberry (*Vaccinium corymbosum*) modulates peripheral arterial dysfunction induced by acute cigarette smoking [97]. Moreover, resveratrol prevents cigarette smoking-induced ROS and carbonyl formation in human keratinocytes [98]. Apple polyphenol, the main sources of flavonoids, not only significantly and dose-dependently reduced cigarette smoking-induced accumulation of inflammatory cells and gene/protein expression of proinflammatory factors but also reversed oxidative stress in the lungs via P38 mitogen-activated protein kinase (MAPK) signaling pathway [99]. p38*α* MAPK was first recognized for its role in inflammation by regulating the biosynthesis of proinflammatory cytokines, namely, IL-1 and TNF-*α*, in endotoxin-stimulated monocytes [100]. Tea polyphenols can antagonize cigarette smoking-induced airway epithelial cell apoptosis through the effective removal of ROS, thereby promoting Bcl-2 mRNA expression and inhibiting the expression of Bax mRNA [101].

### 3.2. Polyphenols and Oxidative Stress Associated with Pathology of Atherosclerosis

#### 3.2.1. Endothelial Dysfunction

Lining the interior surface of vessel cells, endothelial cells could play an essential role in homeostasis, immune, inflammation, cell adhesion, and regulation of thrombosis and fibrinolysis [50, 102]. They maintain vascular tone by regulating various vasodilator factors such as NO and vasoconstrictive factors such as endothelin-1 (ET-1).

Endothelial dysfunction is often associated with increased oxidative stress [50] and impaired mitochondrial activity [103]. Oxidative stress would alter endothelial signal transduction and redox-regulated transcription factors to increase vascular endothelial permeability and catalyze leukocyte adhesion [104]. Shortly after then, endothelial dysfunction can lead to pathologic process of atherosclerosis [105, 106].

**Figure 1:**
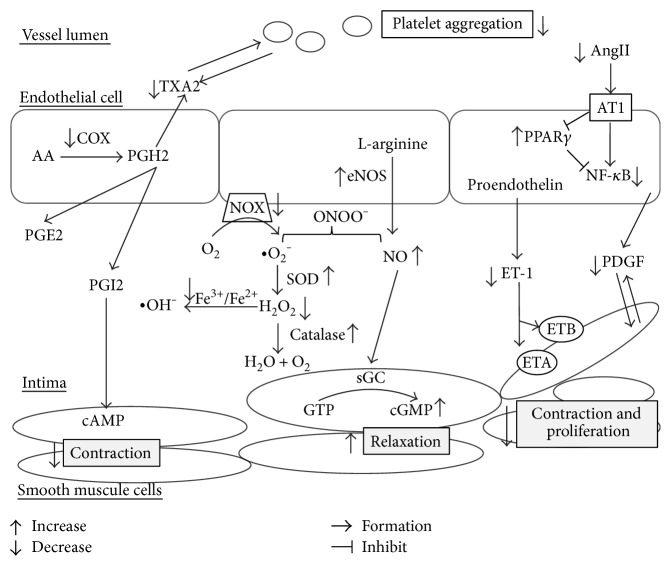
Effects of polyphenols in endothelial cells and smooth muscle cells. AA: arachidonic acid; COX: cyclooxygenase; PGE2/H2/I2: prostaglandin E2/H2/I2; TXA2: thromboxane A2; eNOS: endothelial nitric oxide synthase; NO: nitric oxide; ET-1: endothelin-1; ETA/B: endothelin A/B receptor; LDL: low-density lipoprotein; PDGF: platelet-derived growth factor; NOX: NADPH oxidase; SOD: superoxidase dismutase; H_2_O_2_: hydrogen peroxide; GTP: guanosine triphosphate; sGC: soluble guanylate cyclase; cGMP: cyclic guanosine monophosphate; AngII: angiotensin II; AT1: angiotensin II receptor type 1; PPAR*γ*: peroxisome proliferator-activated receptor *γ*; NF-*κ*B: nuclear factor kappa B.

Some articles showed hydroxytyrosol and the polyphenol extract from extravirgin olive oil may reverse the decreased endothelial NO synthase phosphorylation, intracellular NO levels, and increased ET-1 synthesis by the stimulation of ROS production with high glucose and linoleic and oleic acid levels. In addition, they also could revert the reduced NO and increased ET-1 levels by acetylcholine inducing with high glucose and free fatty acids [107]. Dark chocolate with high flavonoid consumption may ameliorate endothelium-dependent dilation of the brachial artery and increase plasma epicatechin concentrations in healthy adults [108]. Red wines and grapes could elevate the level of cyclic GMP which is the mediator of nitric oxide-induced vascular smooth muscle relaxation through exhibiting endothelium-dependent relaxation of blood vessels and increasing biological activity of NO [109] (Figure 1).

#### 3.2.2. Oxidized Low-Density Lipoprotein

The oxidation of low-density lipoprotein (LDL) is a complex process in which both the protein and the lipids undergo oxidative changes and form complex products. Oxidative stress and LDL oxidation might play a vital role in atherosclerosis, which has been studied for several years. Strong evidence about the close relationship between OxLDL and atherosclerosis exists [110–112].

All these reactions are oxidative in nature, and they are not uniformly amenable to inhibition by traditional antioxidants. Vitamin E or simple phenols, such as tyrosine or estradiol, actually enhance peroxidase-mediated LDL oxidation. Antioxidative ability and concentrations of antioxidants are positively related [113]. Maiolino et al. reviewed the results of randomized clinical trials employing antioxidants and reported that despite demonstrating no benefits in healthy populations, antioxidant use suggests a benefit in high-risk patients [114].

**Figure 2:**
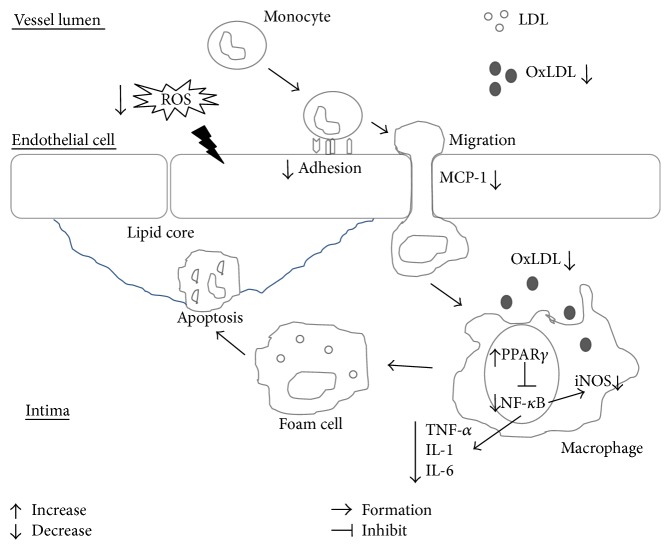
Effects of polyphenols in LDL and inflammatory process with monocytes and macrophages. ROS: reactive oxygen species; LDL: low-density lipoprotein; OxLDL: oxidized low-density lipoprotein; MCP-1: monocyte chemoattractant protein 1; iNOS: inducible nitric oxide synthase; TNF-*α*: tumor necrosis factor-*α*; IL-1: interleukin-1; IL-6: interleukin-6; PPAR*γ*: peroxisome proliferator-activated receptor *γ*; NF-*κ*B: nuclear factor kappa B.

The term “French paradox” is first used in the newsletter of the International Organization of Vine and Wine in 1986. It says a high-fat diet with a low incidence of coronary atherosclerosis is due to moderate consumption of red wine. In 1991, Serge Renaud, a scientist from Bordeaux University, France, made a series of studies that strongly support the result [115]. In vitro studies of phenolic substances in red wine and normal human LDL showed that red wine inhibits copper-catalyzed LDL oxidation [116]. An in vitro study by Chen et al. implied that (−)-epicatechin gallate-enriched *Hibiscus sabdariffa* leaf polyphenols upregulate the autophagic pathway, which in turn led to reduction of OxLDL induced by human umbilical vein endothelial cell injury and apoptosis [117]. Suzuki-Sugihara et al. found that green tea catechins are incorporated into LDL particles in nonconjugated forms after the incubation of green tea extract and reduced the oxidizability of LDL [118] (Figure 2).

#### 3.2.3. Vascular Smooth Muscle Cell Proliferation

VSMCs contribute to the pathogenesis of atherosclerotic lesions; their proliferation and migration are critical events for progressive intimal thickening and arterial wall sclerosis development. Platelet-derived growth factor (PDGF) is the most potent chemotactic and mitogenic agent for VSMCs at the atherosclerotic lesions. They are released by platelets, endothelial cells, and VSMCs themselves. PI3K [119] and MAPK pathway [120, 121] activation as a response to PDGF is implicated in VSMC motility.

Attenuation of the signals leading to VSMC proliferation and migration could also be a consequence of PDGF *β* receptor inhibition by red wine polyphenols [122]. Polyphenol fractions of different molecular weights, for example, 200–400 for monomeric components (anthocyanosides, catechins, and flavonoids) and 1600–2000 for oligomeric proanthocyanidins, showed similar antiproliferative effects [123]. VSMC migration and matrix metalloproteinase-2 (MMP-2) activation are related to atherosclerosis formation. Pterostilbene, a polyphenol compound in blueberries, inhibits VSMC migration, and MMP-2 activation could be mediated via Erk1/2 phosphorylation [124]. Brain-derived neurotrophic factor (BDNF) is considered an essential element in maintaining stable cerebral blood flow. Resveratrol increases serum BDNF concentrations and reduces VSMC contractility via a NOS-3-independent mechanism [125].

As discussed in Section 3.1.1, AngII-induced production of inflammatory factors and VSMC proliferation play a vital role in the progression of atherosclerotic plaques. The activation of PPAR*γ* effectively attenuates AngII-induced inflammation and intercellular ROS production. Curcumin downregulates the expression of p47phox (a key subunit of NADPH oxidase), inhibits the expression of IL-6 and TNF-*α*, decreases the production of NO, and suppresses the proliferation of VSMCs by elevating PPAR*γ* activity and suppressing oxidative stress [126] (Figure 1).

#### 3.2.4. Inflammatory Process with Monocytes, Macrophages, and T Lymphocytes

Macrophages play a key role in atherogenesis through their proinflammatory action, which involves the production of IL-1 and tumor necrosis factor, and following more specific adaptive responses mediated by T cells [127, 128]. Macrophage cells pretreated with TF3 could reduce cell-mediated LDL oxidation by decreasing superoxide release from macrophages [33].

The unsaturated aldehyde acrolein is proatherogenic. Acrolein exposure increases intracellular oxidative stress and stimulates cholesterol and triglyceride accumulation via enhanced biosynthesis rates and overexpression of key regulators of cellular lipid biosynthesis. Acrolein also demonstrates a major shift in the gut microbiota composition wherein a significantly increased prevalence of Ruminococcaceae and Lachnospiraceae, of which the *Coprococcus* genus was significantly and positively correlated with serum, aortic, and macrophage lipid levels and peroxidation, was noted. These proatherogenic effects of acrolein on serum, aortas, macrophages, and the gut microbiota were substantially abolished by pomegranate juice [129]. Polyphenol-rich pomegranate juice inhibits macrophage foam cell formation. Moreover, Sarkar et al. reported that ellagic acid, a phenolic lactone, inhibits tautomerase activity of human macrophage migration inhibitory factor (MIF) by inhibiting MIF-induced NF-*κ*B nuclear translocation [130]. Hydroxytyrosol, a major olive oil antioxidant polyphenol in cardioprotective Mediterranean diets, could suppress MMP-9 and COX-2 activity and expression in activated human monocytes via PKC*α* and PKC*β*1 inhibition [131]. Short-term oral administration of polyphenol-rich extract resulted in a modest anti-inflammatory effect in subjects with clustered metabolic risk factors by reducing inflammatory chemokines, for example, monocyte chemoattractant protein 1, and MIF [132]. Ford et al. compared the effects of 31 polyphenols and 6 polyphenol mixtures on proinflammatory cytokine release by Jurkat T lymphocytes and revealed that resveratrol, isorhamnetin, curcumin, vanillic acid, and specific polyphenol mixtures reduced proinflammatory cytokine release from T lymphocytes. Therefore, polyphenols may decrease proinflammatory mediators especially in chronic inflammation [133] (Figure 2).

#### 3.2.5. Platelet Aggregation

Platelet could maintain the hemostasis of the circulatory system [134]. The major platelet activation pathways mediated by agonists involve the arachidonic acid pathway, adenosine diphosphate (ADP) pathway, serotonin pathway, and NO pathway, and the action of free radicals on molecules is involved in platelet aggregation [135].

Polyphenols, such as resveratrol, have antithrombotic effects, which could be attributed to reduced susceptibility to platelet activation and aggregation, reduced synthesis of prothrombotic mediators (eicosanoid synthesis), and decreased gene expression of tissue factor. Resveratrol has been shown to inhibit, in a concentration-dependent manner, platelet aggregation induced by collagen, ADP, and thrombin. Mattiello et al. compared the effect of pomegranate juice and that of the polyphenol-rich extract from pomegranate fruit on platelet aggregation, calcium mobilization, thromboxane A2 production, and hydrogen peroxide formation induced by collagen and arachidonic acid. Both the pomegranate juice and extract reduced all platelet responses, with the latter showing a stronger effect [136]. Other flavonoids have antiplatelet aggregation effects mainly through the inhibition of the arachidonic acid-based pathway [134].

**Figure 3:**
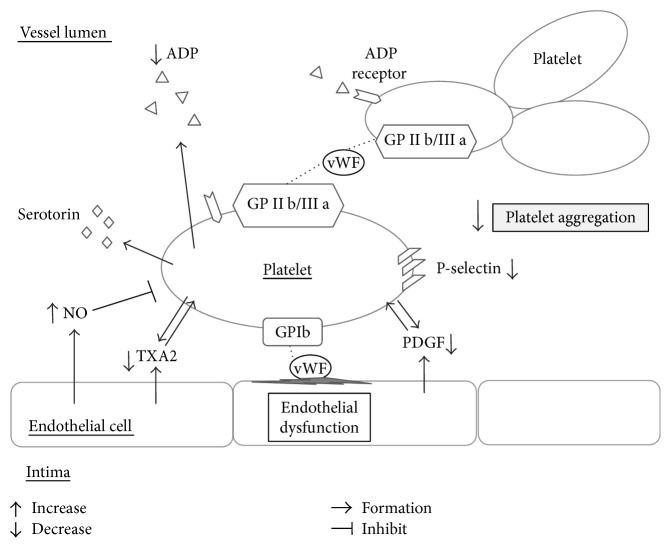
Effects of polyphenols in platelets. ADP: adenosine diphosphate; NO: nitric oxide; TXA2: thromboxane A2; GP1b: glycoprotein Ib; GPIIb/IIIa: glycoprotein IIb/IIIa; vWF: Von Willebrand factor; PDGF: platelet-derived growth factor.

**Figure 4:**
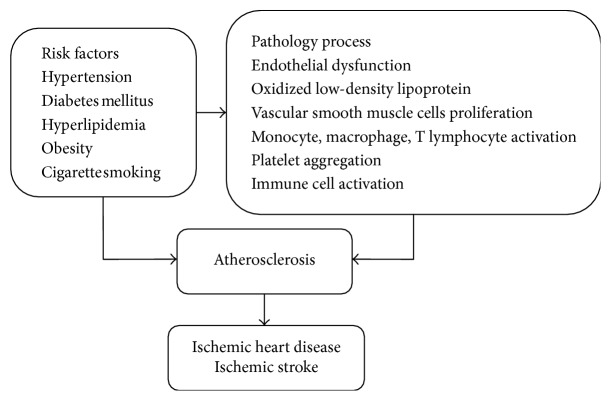
Risk factors and pathology process of atherosclerosis leading to ischemic heart disease or ischemic stroke.

Cocoa and dark chocolate have been shown to prevent platelet aggregation by reducing ADP-, adrenaline-, and epinephrine-induced glycoprotein IIb/IIIa (GPIIb/IIIa) membrane activation; ADP-induced P-selectin membrane expression; and phospholipase A2 (PLA2) and cyclooxygenase activity [137–140]. One trial found that in smokers, dark chocolate dose-dependently inhibits platelet function by lowering oxidative stress. The platelet ROS, 8-iso-PGF2*α*, and NOX2 activation were significantly decreased after dark chocolate consumption [141] (Figures 3 and 4) (Table 1).

### 3.3. Clinical Evidence of Polyphenols and Oxidative Stress in Atherosclerosis-Related Ischemic Heart Disease and Stroke

#### 3.3.1. Polyphenols and Oxidative Stress Associated with Atherosclerosis

Isoflavone in soybeans has antiatherosclerotic property to reduce risk of coronary artery disease and stroke in women [142], but not in men [143]. Interestingly, a randomized controlled trial in the USA showed that isoflavone soy protein supplementation did not significantly reduce subclinical atherosclerosis progression in postmenopausal women but could possibly reduce subclinical atherosclerosis in women at low risk for CVD who were <5 years postmenopausal [144].

A prospective study of forty healthy volunteer women consumed 200 g of açai (one polyphenol-rich fruit which is native to the Brazilian Amazon region) pulp/day for 4 weeks and the result showed açai consumption increased the transfer of cholesteryl esters to high-density lipoprotein and decreased ROS and OxLDL [145].

Cardio-ankle vascular index reflects arterial stiffness which related to atherosclerosis [146]. In a double-blind, randomized, placebo-controlled study, 50 patients with type 2 diabetes mellitus received supplement of a 100 mg resveratrol tablet or placebo daily for 12 weeks. After resveratrol consumption, systolic blood pressure and cardio-ankle vascular index significantly decreased [147].

Plasminogen activator inhibitor type 1 levels are associated with thrombus formation and increased risk of atherosclerosis [148]. One prospective study about nineteen healthy young volunteers, who received oral polyphenol-rich rosemary extracts for 21 days, revealed oral rosemary extracts supplementation improved serum plasminogen activator inhibitor type 1 activity and endothelial dysfunction [149].

#### 3.3.2. Polyphenols and Oxidative Stress Associated with Ischemic Heart Disease

Lekakis et al., in his randomized controlled study of 30 male patients with coronary heart disease, demonstrated that grape polyphenol extract increases flow-mediated dilatation, peaking at 60 min, which was significantly higher than the baseline values or than that of water intake (placebo) [150]. A double-blinded, placebo-controlled, randomized, 3-month study evaluated the efficacy of resveratrol treatment in 40 Caucasian postmyocardial infarction (MI) patients with coronary artery disease. The resveratrol group received 10 mg resveratrol capsule daily for 3 months. Results showed that resveratrol improved left ventricle diastolic function and endothelial function, lowered LDL cholesterol level, and exhibited protection against unfavorable hemorheological changes in patients with coronary artery disease [151]. Several population studies reported an inverse association between flavonoid intake and risk of coronary disease [152–154].

Green tea polyphenols can inhibit H_2_O_2_^−^ induced oxidative stress through the Akt/GSK-3*β*/caveolae pathways in cardiac cells. They could prevent the activation of NF-*κ*b and the inhibition on PI3K/Akt signaling for the acute MI stress. Moreover, green tea polyphenols also could improve mitochondria dysfunction associated with alterations of lipid metabolism, the adaptor 14-3-3 *ε* protein signaling, and chaperone-induced stress response during post-MI remodeling [155].

In a UK women's cohort study, total fruit intake, especially polyphenol-rich fruit group such as grapes and citrus, was associated with lower risk of CVD and coronary heart disease mortality, with a 6-7% risk reduction for every 80 g/day portion consumed [156]. The other results of the PREDIMED multicenter, randomized, primary prevention trial noted that the MeDiet supplemented with nuts, which is rich in unsaturated fat and polyphenols, can be a sustainable and ideal diet for cardiovascular disease prevention [157].

#### 3.3.3. Polyphenols and Oxidative Stress Associated with Ischemic Stroke

According to the WHO, cerebrovascular diseases are the second leading cause of death worldwide and the major cause of disability in adults [158]. Stroke represents 3-4% of the healthcare spending in developed countries [159]. Hence, early protection that would minimize the damage is crucial. The key factor mediating stroke-related damage is oxidative stress. Nutritional intervention, such as polyphenol-enriched diets, has been proposed as preventive and therapeutic agents.

Resveratrol provides protection from cerebral ischemic injury by regulating the expression of silent mating type information regulation 2 homolog 1 (SIRT1). Wan et al. proved that resveratrol provides neuroprotection by inhibiting phosphodiesterase and regulating the cAMP, AMPK, and SIRT1 pathways, which reduces ATP energy consumption during ischemia [160]. Recent findings in animal models and humans showed that polyphenols may have a role in regulating neurotrophin levels, particularly nerve growth factor (NGF) and BDNF, suggesting that polyphenols may also have protective effects through the potentiation of neurotrophin action. NGF and BDNF also act in glucose and energy metabolism and in pancreatic beta cell and cardiovascular homeostasis as metabotropic factors [161]. Salvianolic acid is an active polyphenol component in Danshen (*Salvia miltiorrhiza*) against ischemia/reperfusion injury, and we explored whether the neuroprotection was dependent on mitochondrial connexin43 via the PI3K/Akt pathway. Our previous population-based studies demonstrated that Danshen is the most common herbal drug used to treat ischemic stroke [162].

Wang et al. published a meta-analysis confirming that diets rich in flavonols (intake of 20 mg/day) was associated with a 14% decrease in the risk of developing stroke, specifically among men [163]. Goetz et al. reported the association between flavonoid intake and incident ischemic stroke in a biracial, national cohort using updated flavonoid composition tables and assessed the differences in flavonoid intake by sex, race, and region of residence. The result revealed that greater consumption of flavanones was inversely associated with incident ischemic stroke [164]. We also noted that *Salvia miltiorrhiza* is rich in polyphenol with antioxidant effects by inhibiting oxidases, the production of superoxide, the oxidative modification of low-density lipoproteins, and ameliorating mitochondrial oxidative stress in aging-associated cardiovascular diseases and stroke [165].

## 4. Conclusion

In the result of the study, polyphenol or polyphenol-rich diets exhibit antioxidative and anti-inflammation effects. Polyphenols reduce ROS production through inhibiting oxidases, reducing the production of superoxide, inhibiting OxLDL formation, inhibiting VSMC proliferation and migration, reducing platelet aggregation, and ameliorating mitochondrial oxidative stress. Polyphenol consumption also improves developing into hypertension, diabetes mellitus, hyperlipidemia, and obesity. However, in accordance with in vitro and in vivo laboratory evidence, well-designed clinical studies are necessary to confirm the efficacy of polyphenols in the treatment of atherosclerosis-related IHD and stroke.

## Figures and Tables

**Table 1:** Mechanisms of polyphenols in preventing atherosclerosis formation.

Pathology of atherosclerosis	Polyphenols/polyphenol-rich food	Preventing mechanism	Reference
Endothelial dysfunction	Hydroxytyrosol and EVOO polyphenol extract	↑ eNOS phosphorylation, ↑ NO ↓ ET-1 synthesis ↓ ROS	[107]
	High- versus low-dose flavonoid dark chocolate (213 mg versus 46 mg procyanidins)	↑ endothelium-dependent flow-mediated dilation of the brachial artery ↑ plasma epicatechin concentrations ↔ LDL oxidation, total antioxidant capacity, 8-isoprostanes, blood pressure, lipid parameters, body weight, or body mass index	[108]
	Red wines and grapes	↑ NO activity ↑ cGMP ⊕ vascular smooth muscle relaxation	[109]

OxLDL	Red wine	⊝ copper-catalyzed oxidation of LDL	[116]
	(−)-Epicatechin gallate-enriched *Hibiscus sabdariffa* leaf polyphenols	↓ OxLDL-dependent apoptosis	[117]
	Green tea catechins	Incorporated into LDL particles in nonconjugated forms ↓ oxidizability of LDL	[118]

VSMC proliferation	Red wine	⊝ inhibition of PDGF *β* receptor ↓ VSMC proliferation and migration	[122]
	Pterostilbene, polyphenol compound in blueberries	↓ VSMC migration ⊝ MMP-2 activation via Erk1/2 phosphorylation	[124]
	Resveratrol	↑ serum BDNF concentrations ↓ VSMC contractility via a NOS-3-independent mechanism	[125]
	Curcumin	↓ expression of p47phox ⊝ expression of IL-6 and TNF-*α* ↓ iNOS activity, ↓ NO ⊝ VSMC proliferation ↑ PPAR*γ* activity ⊝ oxidative stress ↓ AngII-induced inflammatory responses	[126]

Monocyte/macrophage and T lymphocytes inflammatory process	Tea flavonoids (theaflavin digallate, theaflavin, epigallocatechin gallate, epigallocatechin, and gallic acid)	↓ cell-mediated LDL oxidation ↓ macrophages release superoxide and iron ions	[33]
	Pomegranate juice	⊝ acrolein increases macrophage lipid accumulation and alters the gut microbiota composition	[129]
	Ellagic acid	⊝ tautomerase activity of human macrophage MIF ⊝ NF-*κ*B nuclear translocation	[130]
	Polyphenol-rich extract	↓ MCP-1 ↓ macrophage MIF	[132]
	Resveratrol, isorhamnetin, curcumin, vanillic acid, and specific (poly)phenol mixtures	↓ IL-6, interferon-*γ* induced protein 10 and TNF-*α* release	[133]

Platelet aggregation	Pomegranate juice or the polyphenol-rich extract from pomegranate fruit	⊝ collagen- and arachidonic acid-induced platelet aggregation ↓ collagen- and arachidonic acid-induced calcium mobilization ↓ thromboxane A2 production ↓ H_2_O_2_ formation	[136]
	Cocoa and dark chocolate	↓ ADP-, adrenaline- and, epinephrine-induced GPIIb/IIIa membrane activation ↓ ADP-induced P-selectin membrane expression ↓ PLA2 and COX activity ↓ ROS, 8-iso-PGF2*α*, and NOX2 activation	[137–141]

↑: increase; ↓: decrease; ↔: no change; ⊝: inhibit; ⊕: promote. EVOO: extra virgin olive oil; eNOS: endothelial nitric oxide synthase; NO: nitric oxide; ET-1: endothelin-1; LDL: low-density lipoprotein; ROS: reactive oxygen species; Erk: extracellular-signal-regulated kinase; PDGF: platelet-derived growth factor; VSMCs: vascular smooth muscle cells; MMP-2: matrix metalloproteinase-2; BDNF: brain-derived neurotrophic factor; MIF: migration inhibitory factor; MCP-1: monocyte chemoattractant protein 1; H_2_O_2_: hydrogen peroxide; ADP: adenosine diphosphate; GPIIb/IIIa: glycoprotein IIb/IIIa; PLA2: phospholipase A2; COX: cyclooxygenase; 8-iso-PGF2*α*: 8-isoprostane-prostaglandin F2*α*; NOX2: NADPH oxidase 2.

## Abbreviations

IHD: Ischemic heart disease
ROS: Reactive oxygen species
•O_2_^−:^: Superoxide radicals
H_2_O_2:_: Hydrogen peroxide
NO: Nitric oxide
eNOS: Endothelial nitric oxide synthase
iNOS: Inducible nitric oxide synthase
OxLDL: Oxidized low-density lipoprotein
LDL: Low-density lipoprotein
VSMCs: Vascular smooth muscle cells
NOS: Nitric oxide synthase
AngII: Angiotensin II
NADPH: Nicotinamide adenine dinucleotide phosphate
NF-*κ*B: Nuclear factor kappa B
NOX: NADPH oxidase
LPS: Lipopolysaccharide
PPAR*γ*: Proliferator-activated receptor gamma
MnSOD: Manganese superoxide dismutase
VEGF: Vascular endothelial growth factor
FAS: Fatty acid synthase
EGF: Epidermal growth factor
PI3K: Phosphatidylinositol-3-kinase
Akt: Protein kinase B
AMPK: AMP-activated protein kinase
TF3: Theaflavin-3,3′-digallate
FoxO3A: Forkhead box O3A
WHO: World Health Organization
BBB: Blood-brain barrier
Nrf2: Nuclear factor erythroid 2-related factor
IL-1: Interleukin-1
TNF-*α*: Tumor necrosis factor-*α*
MAPK: Mitogen-activated protein kinase
ET-1: Endothelin-1
PDGF: Platelet-derived growth factor
MMP-2: Matrix metalloproteinase-2
BDNF: Brain-derived neurotrophic factor
MIF: Migration inhibitory factor
MCP-1: Monocyte chemoattractant protein 1
ADP: Adenosine diphosphate
GPIIb/IIIa: Epinephrine-induced glycoprotein IIb/IIIa
PLA2: Phospholipase A2
MI: Myocardial infarction
CVD: Cardiovascular disease
MeDiet: Mediterranean diet
EVOO: Extravirgin olive oil
SIRT1: Silent mating type information regulation 2 homolog 1
NGF: Nerve growth factor.
